# Identification of New KRAS G12D Inhibitors through Computer-Aided Drug Discovery Methods

**DOI:** 10.3390/ijms23031309

**Published:** 2022-01-24

**Authors:** Apoorva M. Kulkarni, Vikas Kumar, Shraddha Parate, Gihwan Lee, Sanghwa Yoon, Keun Woo Lee

**Affiliations:** 1 Department of Bio and Medical Big Data (BK4 Program), Division of Life Science, Research Institute of Natural Science, Gyeongsang National University, 501 Jinju-daero, Jinju 52828, Korea; apoorvamk19@gmail.com (A.M.K.); vikaspathania777@gmail.com (V.K.); jsyoon0517@gmail.com (S.Y.); 2 Division of Applied Life Science, Plant Molecular Biology and Biotechnology Research Center, Gyeongsang National University, 501 Jinju-daero, Jinju 52828, Korea; parateshraddha@gmail.com (S.P.); pika0131@naver.com (G.L.)

**Keywords:** KRAS, in silico, pharmacophore, virtual screening, molecular docking, molecular dynamics simulations

## Abstract

Owing to several mutations, the oncogene Kirsten rat sarcoma 2 viral oncogene homolog (KRAS) is activated in the majority of cancers, and targeting it has been pharmacologically challenging. In this study, using an in silico approach comprised of pharmacophore modeling, molecular docking, and molecular dynamics simulations, potential KRAS G12D inhibitors were investigated. A ligand-based common feature pharmacophore model was generated to identify the framework necessary for effective KRAS inhibition. The chemical features in the selected pharmacophore model comprised two hydrogen bond donors, one hydrogen bond acceptor, two aromatic rings and one hydrophobic feature. This model was used for screening in excess of 214,000 compounds from InterBioScreen (IBS) and ZINC databases. Eighteen compounds from the IBS and ten from the ZINC database mapped onto the pharmacophore model and were subjected to molecular docking. Molecular docking results highlighted a higher affinity of four hit compounds towards KRAS G12D in comparison to the reference inhibitor, BI-2852. Sequential molecular dynamics (MD) simulation studies revealed all four hit compounds them possess higher KRAS G12D binding free energy and demonstrate stable polar interaction with key residues. Further, Principal Component Analysis (PCA) analysis of the hit compounds in complex with KRAS G12D also indicated stability. Overall, the research undertaken provides strong support for further in vitro testing of these newly identified KRAS G12D inhibitors, particularly Hit1 and Hit2.

## 1. Introduction

RAS genes (KRAS, HRAS, and NRAS) belong to the RAS family of small GTPases. They cycle between an active GTP-bound state and an inactive GDP-bound state, and hence function as molecular switches, depending on extracellular signals. The transition from active to inactive form involves the changes in the conformation which is catalyzed by guanine nucleotide exchange factors (GEFs) and GTPase-activating proteins (GAPs) [1]. GEFs, primarily Son of Sevenless (SOS) homologs SOS1 and SOS2 help in the exchange of bound nucleotide GDP into GTP [2]. GAPs aid in enhancing the intrinsic weak RAS GTPase activity, thus catalyzing RAS inactivation [3]. Among the RAS family, KRAS is widely mutated, with activating mutations in KRAS witnessed in nearly 30% of cancers. In RAS protein, three major hotspots, G12, G13, and Q61, have been reported in the literature, where G12 comprises 83% of all KRAS mutations followed by G13 (14%) and Q61 (2%) [4]. Various G12 mutations such as G12D, G12V, G12C, G12A, G12S, and G12R have been reported [5]; however, G12D mutation is one of the most common [6,7] and is the focus of our research. The common strategies adopted for anti-RAS drug development can be broadly classified into direct and indirect approaches. Direct approaches include compounds that bind directly to RAS and interrupt the interactions between RAS and GEFs/effectors. Indirect approaches include: (1) inhibiting proteins such as farnesyltransferase or PDEδ that promote the association of RAS with plasma membrane; (2) targeting proteins involved in RAS downstream signaling; (3) inhibiting synthetic lethal interaction partners for mutant RAS and (4) targeting processes that are RAS regulated, for example, macropinocytosis and autophagy [7]. Disappointing clinical trial results from the indirect approaches have failed to offer any RAS drugs [8], hence, targeting RAS genes directly has been considered as a viable approach. Relatively extensive research has been conducted on identifying inhibitors for KRAS G12C, as the mutant cysteine would assist in irreversible binding to small molecules [7,9,10]. With Sotorasib obtaining its FDA approval in May 2021 [10], a bright outlook is presented among KRAS researchers. Although the approval is a milestone in the KRAS battlefield, with different mutations presenting different tumor environments, the war continues. MRTX1133 [11], a selective G12D inhibitor developed by Mirati Therapeutics, targets both active and inactive KRAS states; however, the mechanism of action has not been published yet and the drug will most likely enter clinical trials in early 2022 [7,12]. A cyclic peptide–KD2 showing binding to active state KRAS-G12D was identified [12]. Nonetheless, owing to permeability issues, the compound is stated to require further development [6]. Another cyclic peptide, KS-58, which disrupts KRAS G12D interactions with SOS1 and BRAF, was identified, with suggestions for improving its pharmacokinetic profile and dosage put forth by the authors [13]. Several computational groups are also working on identifying KRAS G12D inhibitors and the field seem optimistic. Although first described as a proto-oncogene nearly 40 years ago [14], the first FDA approval has come only after four decades. Early on, a high affinity for GTP and lack of druggable pockets by virtue of its smooth surface were some of the reasons for the failure to discern a KRAS inhibitor [11,12,13,14]. However, as a consequence of the discovery of two pockets on the surface of RAS, namely, switch I and switch II, the undruggable KRAS has been shown to be druggable indeed [3,15,16,17,18]. It is important to highlight that the switches have different amino acid residue definitions. Some researchers define switch I with residue numbers ranging from 25–40 [15], 30–37 [16], 30–40 [17], and 30–38 [18]. The beginning of switch II falls between residues 58–60 and ends between 67–76 in different research papers [19]. In our research, we have adopted the residue definition of Shokat et al. [18], i.e., switch I spanning residues 30–38 and switch II spanning residues 60–76. These two switches are held by γ-Phosphate of GTP in a manner amenable for interactions with SOS1 and SOS2 and downstream effectors. Several inhibitors that bind to a region close to switch II were identified, such as ARS-853 [20], tetrahydropyridopyrimidines [21], MRTX849 [22], and AMG510 [23]., However, their covalent nature restricts the usage to only G12C mutations and cancers, with other KRAS mutations such as G12D, G12V, and Q61H lacking the reactive cysteine to be an effective target for these covalent KRAS inhibitors [24]. Recently, Kessler et al. discovered BI-2852 as a direct inhibitor of KRAS that targets the region between switch I and II. BI-2852 was shown to form polar interactions with Glu37, Ser39, and Asp54, and by doing so, it blocks the interaction of KRAS with SOS and also reduced pERK and pAKT levels [3]. It was observed that BI-2852 bound to GTP-KRAS G12D with a nanomolar binding affinity (IC_50_ of 450 nM). Therefore, by using BI-2852 as a chemical probe, the intent of the research was to computationally identify chemical compounds exhibiting higher KRAS G12D binding capabilities. Our study encompasses a generation of pharmacophore models from previously reported KRAS G12D inhibitors to retrieve important features for KRAS G12D inhibition followed by mapping of potential compounds from drug-like databases. Thereafter, molecular docking and molecular dynamics simulations were implemented to identify probable KRAS G12D inhibitors. 

## 2. Results

### 2.1. Common Feature Pharmacophore Generation

**Figure 1:**
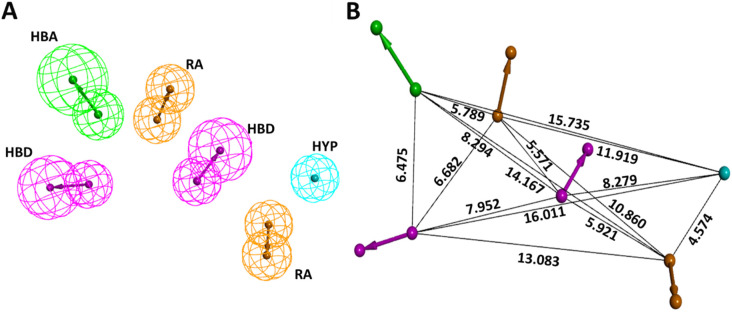
Selected common feature pharmacophore specifics. (**A**) Essential features for KRAS G12D inhibition. (**B**) Interfeature distance (in Å) among the features.

Prior to the generation of the pharmacophore model, by utilizing the three KRAS G12D inhibitors reported by Kessler et al. [3], we first discerned the important chemical features for their activity. This was accomplished by the “Interaction Generation” tool in DS. Based on the results, hydrogen bond acceptor (HBA), hydrogen bond donor (HBD), ring aromatic (RA), and hydrophobic (HYP) were chosen as a criterion for common feature pharmacophore generation. Ten common feature pharmacophore models were generated. Models 1–8 displayed an identical score of 36.17 and models 9 and 10 each scored 35.96. Further, we also checked for the fit value of our active compounds in models 1–8 and based on higher fit values of 5.99, 2.88, and 2.48 for compound 1, compound 2, and compound 3, respectively, Model 3 seemed promising. The selected pharmacophore model containing 2HBD, 2RA, 1HYP, and 1HBA, along with interfeature distances among them, is shown in Figure 1. The characteristics of all 10 generated common feature pharmacophores are outlined in Table 1.

### 2.2. Pharmacophore Validation

To validate the chosen model, Model 3, a ROC curve was generated. Model 3 identified all three active compounds as true positives, while seven out of eight inactive compounds were identified as true negatives. The sensitivity of Model 3 was 1, thus indicating its robust ability to pick out active KRAS inhibitors. The specificity obtained was 0.87. The ROC determined by the area under curve (AUC) achieved a score of 1, thus indicative of a perfect model. The ROC curve for Model 3 is shown in Figure S1. Although other models displayed perfect sensitivity of 1 by accurately discriminating the active inhibitors from inactive inhibitors, their specificity score indicative of identifying true negatives was less than that of Model 3. Model 10 displayed a specificity and sensitivity score on par with Model 3, however, it displayed a score (35.96) lesser than Model 3 (36.17) (Table 1). Further, a manual assessment of pharmacophore mapping of active and inactive compounds on both models showed Model 3 to be a better fit. Hence, Model 3 was selected. The pharmacophore validation parameters are outlined in Table 2.

### 2.3. Virtual Screening

The compounds from IBS and ZINC were first filtered with Lipinski’s parameters to ensure drug-likeness of potential inhibitor compounds. This resulted in 50,221 and 122,102 compounds from the respective databases. Further, ADMET filtering, based on parameters mentioned in the Material and Methods section were performed for each of the compounds from the two databases. Consequently, 15,603 and 34,329 compounds were obtained from IBS and ZINC, respectively. These compounds were then mapped onto the selected pharmacophore, Model 3. Based on this, 18 compounds from IBS and 10 from ZINC were retrieved. Therefore, a total of 28 compounds were subjected to further molecular docking.

### 2.4. Molecular Docking

The crystal structure of KRAS G12D in complex with BI-2852 (PDB id: 6GJ8) [3] was used for this study. The PDB structure has a resolution of 1.65 Å. To examine the binding affinity of 28 compounds obtained after virtual screening, we docked these compounds at the binding site of the inbound inhibitor, BI-2852, which is used as a reference in this study. Reference demonstrated -CDOCKER energy and -CDOCKER interaction energy of 25.01 kcal/mol and 46.9 kcal/mol, respectively. Hit1, Hit2, and Hit4 displayed greater -CDOCKER energy and -CDOCKER interaction energy than Reference. Hit3 displayed higher -CDOCKER interaction energy and comparable -CDOCKER energy in comparison to Reference. These scores are indicated in Table 3.

Next, we examined the interactions of these ligands with the target protein. Reference demonstrated a total of four hydrogen bonds, with switch I residue Glu37 (one bond), Ser39 (one bond), and Asp54 (two bonds). Hit1 also formed a total of four hydrogen bonds: one each with Leu6, switch I residue Glu37, Ser39, and Asp54 (as a part of a salt bridge). Hit2 formed three hydrogen bonds: one with switch I residue Glu37 and two with Asp54. Hit3 also demonstrated three hydrogen bonds (one each with Lys5, Ser39, and Arg41), while Hit4 demonstrated two hydrogen bonds with switch I residue Gly37. The hydrogen bonds and other interactions between KRAS G12D and hit compounds are demonstrated in Figure S2. These four hits were further subjected to molecular dynamics simulations.

### 2.5. Molecular Dynamics Simulations

Molecular dynamics (MD) simulation is a powerful tool to analyze the dynamic nature of a protein–ligand complex at the atomic level. Accordingly, the best docked pose obtained from molecular docking analyses of reference and hit compounds were subjected to a 100 ns MD simulation with KRAS G12D.

#### 2.5.1. RMSD and RMSF Assessment

**Figure 2:**
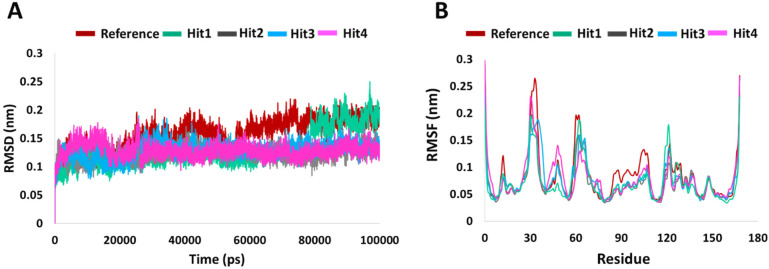
System stability analysis. (**A**) RMSD and (**B**) RMSF profiles of KRAS G12D in complex with reference and hits compounds.

RMSD is indicative of any changes in the atomic position from the initial structure and is a quantitative measure for the stability of the structure.The average backbone RMSD for the four hits was 0.12 nm, with very low standard deviations of 0.01, 0.005, 0.01, and 0.02 among the triplicate MD runs. For the Protein-Reference complex, the average RMSD was 0.15 nm for the three runs. The RMSD profile is shown in Figure 2. The initial increase in the RMSD observed for all hits–protein complexes are due to system adaptability. Minimum deviations (<0.3 nm) [25] were seen for Reference and Hit1. That aside, no major RMSD fluctuations were observed for any of the systems.

To understand the per residue fluctuations upon ligand binding, RMSF calculation were performed. As in previously published results [26,27], in comparison to the whole protein, fluctuations were mainly witnessed around switch I and switch II residues. In comparison to other systems, Hit1 saw a higher and insignificant peak around residue Leu120. Therefore, RMSD and RMSF analyses supported further screening potential of our hits’ compounds.

#### 2.5.2. Binding Dynamics and Molecular Interactions

**Figure 3:**
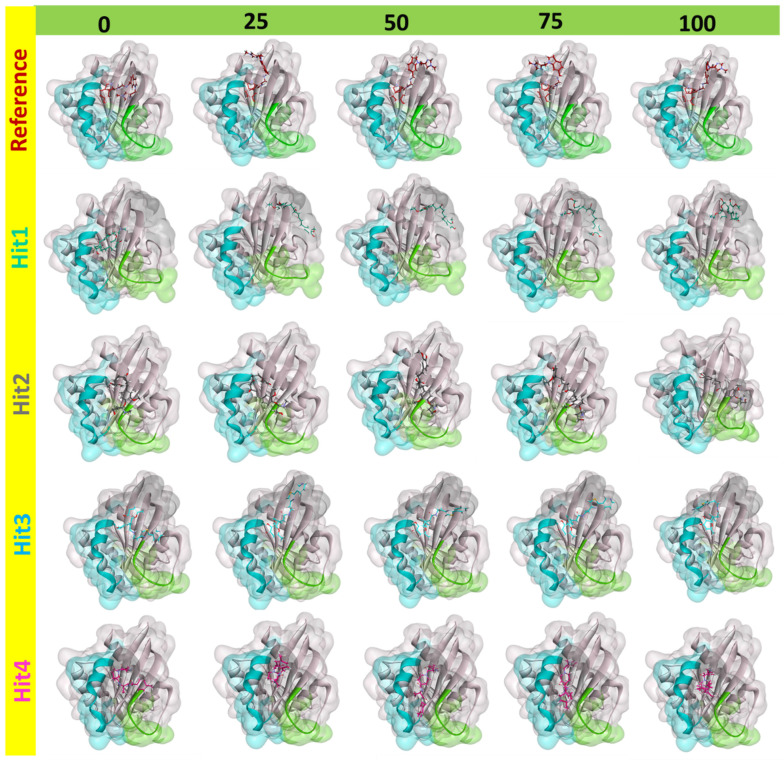
Binding profiles of reference and hits molecules with KRAS G12D at different simulation intervals represented in ns. The switch I and switch II regions of the protein are indicated in green and blue colors, respectively.

Binding profiles of the hit molecules with the protein were determined by capturing and superimposing the snapshots at 0, 25, 50, 75, and 100 ns. The binding profiles are shown in Figure 3. Reference, Hit3 and Hit4 showed similar binding dynamics. Their profiles from 25 ns onwards demonstrated that the compounds had moved closer to switch II residues. On the other hand, Hit2 demonstrated proximity towards switch I. Interestingly, binding proceedings of Hit1 revealed movement away from the two switches, beginning from 25 ns until the end of MD simulation. Overall, this analysis suggests that the hits remain in complex with the protein throughout the 100 ns simulation.

**Figure 4:**
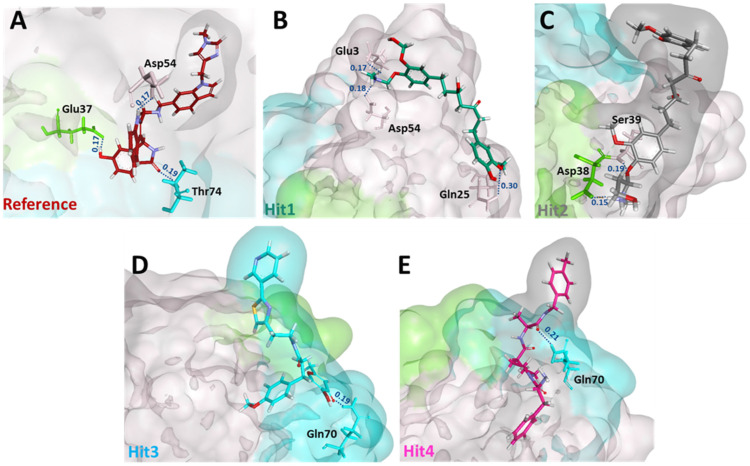
Post MD molecular interactions of reference (**A**) and hit compounds (**B**–**E**) with KRAS G12D. The reference, hit compounds and KRAS residues involved in salt bridge/hydrogen bonding are depicted by the stick model. Residues from switch I and switch II contributing to salt bridge/hydrogen bonds are colored in green and blue, respectively, while residues not part of either switch are colored in salmon. Interatomic distances in nm are indicated in blue above the dotted lines.

Next, we sought to determine the intermolecular bonds between the ligands and the KRAS G12D. Reference displayed hydrogen bonds with Glu37, Asp54, and Thr74. Interestingly, Hit1 demonstrated salt bridges with Glu3 and Asp54, while Hit2 demonstrated salt bridge interaction with Asp38. Additionally, Hit1 and Hit2 also formed hydrogen bonds with Gln25 and Ser39, respectively. Hit3 and Hit4 both interacted with Gln70 via a hydrogen bond. The residues, along with their interatomic distances between bonds, are shown in Figure 4. 

**Figure 5:**
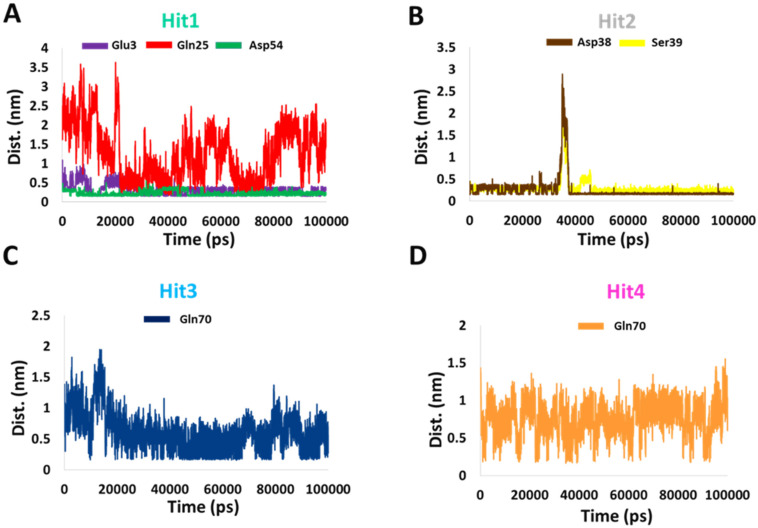
Distance profiles of important residues with (**A**) Hit1, (**B**) Hit2, (**C**) Hit3, and (**D**) Hit4 during the MD simulation.

Subsequently, to determine the stability of these bonds, we calculated the distance between the donor and recipient atoms. The average distance between Hit1 atoms involved in salt bridge formation with Glu3 and Asp54 were 0.3 and 0.2 nm, respectively. The average atomic distance between Hit1′s O18 and HE21 from Gln25 was 1.25 nm, and therefore the stability of this bond is questionable. The average distance between Hit2 H60 atom involved in salt bridge formation with OD2 of Asp38 was 0.25 nm and the average distance between O27 of Hit2 and HN of Ser39 was 0.2 nm. The average distances between the atoms of Hit3 and Hit4 involved in hydrogen bonding with Gln70 were 0.6 and 0.7 nm, respectively. Our distance analysis indicates that Hit1 and Hit2 are likely to form more stable interactions with KRAS G12D than Hit3 and Hit4. The distance profiles are shown in Figure 5.

Additionally, hydrogen bond occupancy, defined as the average number of hydrogen bonds per time frame, were also calculated for these important residues. The percentages of hydrogen bond occupancies are shown in Table 4. With an above 85% rate of formation of hydrogen bonds of Hit1 with Glu3 and Asp54 and Hit2 with Asp38 and Ser39, both Hit1 and Hit2 indicated a very reliable hydrogen-bonding profile. Overall, from the intermolecular interaction analysis of hits with KRAS G12D, it could be inferred that Hit1 and Hit2 might form more stable and consistent interactions than Hit3 and Hit4. Comparison between polar interactions before MD (i.e., the molecular docking) (Figure S2) and after MD (Figure 4) were in agreement with our binding dynamics analysis (Figure 3). These interactions, along with their distance measurements, are shown in Table 5. Hit1 lost its interaction with switch I residue Glu37 and the nearby Ser39. Interaction with Leu6 was lost. However, as a part of the salt bridge, a hydrogen bond was formed with Glu3. Interaction with Asp54 was maintained both before and after MD, although based on the distance of 0.18 nm, after MD, the hydrogen bond with Asp54 was more stable. Hit2, due to its movement toward switch I, formed stable polar interactions with Asp38 and Ser39. On the other hand, Hit3 and Hit4, due to their movement towards switch II, as seen during the binding dynamics (Figure 3), gained hydrogen bond interaction with Gln70. They also lost their interactions with Switch 1 residue Glu37 and residues close to switch I such as Ser39 and Arg41. Besides this, it was also learned that hit molecules and reference from MD formed polar interactions with higher stability, as seen by the reduced distance measurements in Table 5.

Additionally, Reference formed π interactions with Lys5, Val7, Arg41, Leu56, and Met67. Van der Waals interactions were witnessed between Reference and KRAS G12D with switch II residues Gln70, Tyr71, and Gly75 among others. Hit1 formed π-alkyl interactions with Leu52 and Arg41 and van der Waals interactions with residues not falling in either switch I or II regions. Hit2 demonstrated van der Waals interaction with Glu37, Gln70, and Tyr40. Hit3 formed π interactions with Arg41, Leu56, and Tyr71. Hit3 was involved in forming van der Waals interactions with Asp38, Ser39, and Thr74 among others. Hit4 formed π interactions with Lys5 and Leu56, and van der Waals interaction were seen with Glu37, Asp54, and Thr74 among others. A complete list of these interactions is provided in Figure S3.

#### 2.5.3. Binding Free Energy

**Figure 6:**
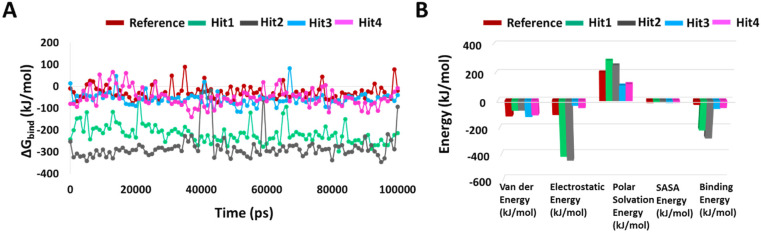
Binding free energy analyses. (**A**) Graphical representation of MM/PBSA estimated binding free energy of the reference and hits compounds with KRAS G12D throughout 100 ns MD. (**B**) Binding free energy decomposition plot.

To determine the binding free energy of small ligands to proteins, the molecular mechanics energies combined with the Poisson–Boltzmann or generalized Born and surface area continuum solvation (MM/PBSA and MM/GBSA) methods are popular approaches [28]. Using “g_mmpbsa”, we calculated the average binding free energies of reference and hit compounds with KRAS G12D every 100th frame of the simulation. The average binding free energy for KRAS G12D in complex with Reference, Hit1, Hit2, Hit3, and Hit4 were −27.888 +/−30.440 kJ/mol, −217.091 +/−41.830 kJ/mol, −276.903 +/−63.029 kJ/mol, −57.011 +/−28.093 kJ/mol, and −51.031 +/−40.608 kJ/mol, respectively. It is worth mentioning that there could be multiple reasons behind the high error estimations, including the execution of single long simulations rather than multiple small runs [28], the number of frames selected [28], and the inclusion of the entire trajectory for analysis. All the hit compounds showed better binding free energies with KRAS G12D than the reference inhibitor, with Hit1 and Hit2 significantly outperforming Hit3 and Hit4. A decomposition analysis of these binding energies indicated that electrostatic energy was the major contributor. The binding free energies, along with the decomposition analysis, are shown in Figure 6.

#### 2.5.4. PCA

**Figure 7:**
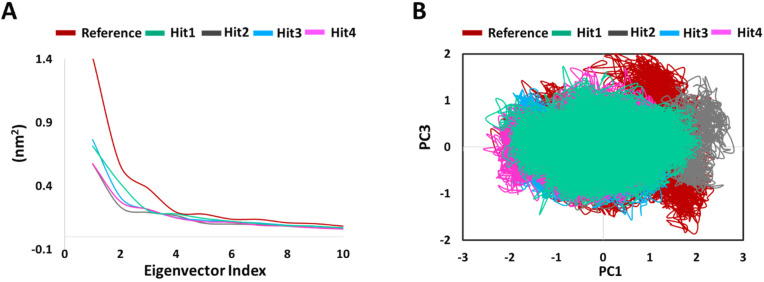
Principal component analysis. (**A**) Eigen values plotted vs eigenvector index, (**B**) A 2D projection plot of reference and hits in complex with KRAS G12D.

PCA was conducted to assess the ligand binding induced correlated motions. The overall significant motion of the protein is always controlled by the first few eigenvectors [29,30]. The top 10 eigenvectors accounted for 70%, 72%, 55%, 60%, and 58% motions for Protein-Reference, Hit1, Hit2, Hit3, and Hit4, respectively. We then plotted the first 10 eigenvectors against their eigenvalues for reference and hits compounds in complex with KRAS G12D (Figure 7A). As expected, the resulting plot indicated that eigenvalues for the first few vectors were higher, with the most important motions in our case contained in the first, second, and third eigenvectors. Figure 7A also demonstrates that values for the first few vectors were higher for Protein-Reference as compared to Protein-Hits, indicating that the binding of Reference leads to greater conformational changes in proteins dynamics as compared to binding of Hits [29]. We generated a 2D projection plot of these components for both the Protein-Reference and Protein-Hits (Figure 7B). Figure 7B demonstrates that the components of our hits’ complexes are less scattered and occupy lesser phase space in comparison to the reference. These observations thus highlight the better integrity of the Protein-Hits complexes.

## 3. Discussion

Long presumed as an undruggable target, the RAS family has always been a problematic target in drug discovery projects. Because of the lack of a well-defined hydrophobic pocket on RAS protein surfaces, initially a lot of research was focused on indirect approaches such as targeting downstream RAS effectors or targeting farnesyltransferase or PDEδ to curb RAS [7]. However, recently, using chemical screens, several compounds were identified that directly bind to the KRAS4B variant and disrupt its function. One such compound is BI-2852, which was discovered as a direct inhibitor of KRAS G12D [3]. BI-2852 binds to a pocket between switch I and II and blocks all GEFs, GAP, and effector interactions, thus leading to downstream signaling inhibition and anti-proliferative effects. In the process of discovering BI-2852, two other compounds, **21** and **22**, were discovered that displayed a nM binding potential to KRAS [3,31]. With the purpose of finding more potent KRAS inhibitors, we conducted this insilico analysis. By utilizing the three compounds (BI-2852, compound **21**, and compound **22**), we first developed a common feature pharmacophore model to discern the potential necessary features for KRAS G12D inhibition. These features included HBD, HBA, RA, and HYP, as shown in Figure 1. Having completed this, we also put forward a limitation that since the three active compounds were very similar in their structure, the pharmacophore might have missed compounds that in reality may be active KRAS G12D inhibitors but possess different features. Additionally, negative results are rarely published and the limited inactive dataset used here might fail to completely delineate the selectivity and sensitivity during the validation of the model [32]. It is also important to take into account that the active compounds may demonstrate their effect via other mechanisms than the intended one, while the inactive compounds may actually interact with the target but, due to poor pharmacokinetic properties, might go undetected [32].

Poor pharmacokinetic profile often leads to the failure of drugs in pre-clinical/clinical trials [33]. Thus, prior assessment of these parameters can help mitigate the economic losses that could occur in later stages. Table S2 lists the drug-likeness and ADMET results of the hit compounds. All identified hits displayed a favorable Lipinski profile and demonstrate ADMET properties in agreement with those previously reported for computationally identified potential cancer drugs [34,35]. The compounds passing the drug assessment tests were then mapped onto the selected pharmacophore model to retrieve compounds that would potentially have the ideal features of KRAS inhibition as demonstrated by selected active KRAS inhibitors.

**Figure 8:**
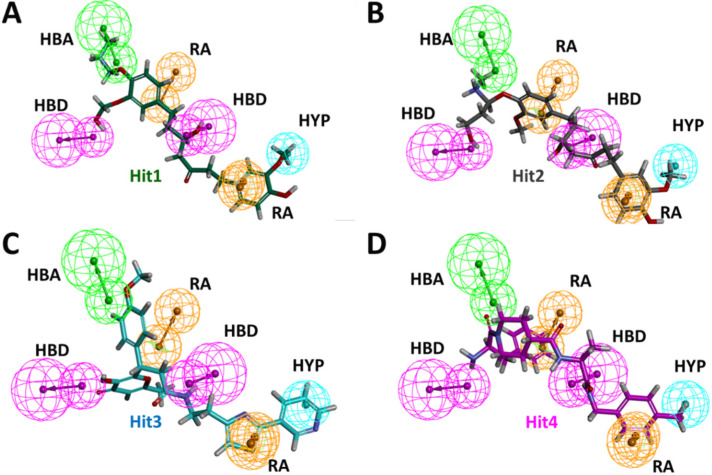
Post MD pharmacophore mapping of hit compounds (**A**) Hit1, (**B**) Hit2, (**C**) Hit3 and (**D**) Hit4.

Binding mechanistic studies of hits through molecular docking indicated that the hit compounds possessed an enhanced affinity to KRAS G12D in comparison to BI-2852 (Table 3). However, because molecular docking fails to provide real-time drug-target interaction and does not take into account the simulated physiological environment, we performed MD simulation. Interaction comparison presented in Table 5 also supports the observation that molecular docking might not always be in agreement with molecular dynamics data. Usually, an RMSD value below 0.3 nm is well accepted [25]. The average backbone RMSD analysis of all our Protein-Hit complexes was below 0.3 nm, thus suggestive of a stable protein–ligand complex (Figure 2A). Additionally, apart from switch I and switch II RMSF fluctuations (Figure 2B) commonly seen in mutated KRAS systems [26,27], no significant RMSF fluctuations were observed. PCA is a powerful tool to gain knowledge on conformational changes in a structure, with many researchers now using PCA to study ligand binding induced protein conformation changes [29,30]. In line with the RMSD and RMSF profiles, the PCA results also displayed fewer correlated motions upon hit molecules binding in comparison to reference. Cumulatively, these analyses indicate that the hit molecules identified might not impart significant structural changes to the KRAS G12D and will form a stable complex with it. As inference from molecular interactions might aid in determining the mechanism of action of a drug, a thorough examination of the interaction pattern of our hit compounds was conducted. The interactions are presented in Figure 4 and Figure S3. Salt bridges are a combination of a hydrogen bond and an ionic bond, and thus are stronger than conventional hydrogen bonds. Hit1 formed salt bridges with Glu3 and Asp54 and formed π- alkyl interaction with Arg41. Asp54 and Arg41 from KRAS have been shown to participate in salt bridges with His911 and Asp910 of SOS, thereby stabilizing the KRAS-SOS complex [36]. Maurer et al. had discovered 4, 6-dichloro-2-methyl-3-aminoethylindole (DCAI), a small molecule inhibitor for KRAS. According to [36], DCAI-induced conformational changes in KRAS disrupted the Asp54-His911 and Arg41-Asp910 salt bridges between KRAS and SOS and contributed to the inhibitory action of DCAI. We argue that Hit1 due to its ability to engage with Asp54 and Arg41 might function in a similar way. In our analysis, Hit2 has demonstrated a salt bridge with Asp38. In previous studies, it has been shown that Asp38 is a critical residue for KRAS-RAF [37] and KRAS-PI3K association [38]. In fact, a reduced binding affinity of KRAS inhibitor- compound 3144 was observed when Asp38, along with Ile36, was mutated [39]. The results from these studies thus highlight the importance of Hit2 in engaging the crucial Asp38 residue. Further, it was also noticed that these salt bridges, with their average distances less than 0.3 nm throughout the simulation runs, were quite stable. It is important to highlight here that these salt bridges were consistent in all three simulation runs, and were the crucial electrostatic energy drivers in the protein–ligand binding. The magnitude of binding free energy could determine how strongly the ligand interacts with the target and thus can directly relate to its potency [40]. The electrostatic interactions formed by the salt bridges discussed above are shown to make major contributions to the remarkable binding free energy of Hit1 (−217.091 +/−41.830 kJ/mol) and Hit2 (−276.903 +/−63.029 kJ/mol) (Figure 6). Additionally, polar interactions with Ser39 were responsible for the increased potency of BI-2852 towards KRAS G12D [3]. While the reference pose of BI-2852 with KRAS after MD displayed no Ser39 bond, Hit2 formed stable hydrogen bonds of distance 0.2 nm with Ser39 throughout the MD run. Hit3 and Hit4 also displayed moderate stability of their hydrogen bond with Gly70, with average distances of 0.6 nm and 0.7 nm, respectively (Figure 7). Pharmacophore mapping of our hits compounds post MD is shown in Figure 8. Hit2 and Hit4 aligned to all the pharmacophore features. Hit1 and Hit3 missed only one feature (RA) and aligned well to all the other features, thus indicating the robustness of our pharmacophore model to choose the active KRAS G12D inhibitors. 

The sequence of the chosen binding site (i.e., the region between switch I and II) is identical in all RAS isoforms [31], thus we were curious to explore the binding affinity of our hit compounds towards HRAS and NRAS. Molecular docking of all four hit compounds with HRAS G12D (PDB id: 6ZJ0) and NRAS Q61R (PDB id: 6ZIZ) revealed their higher inclination towards the protein than the inbound inhibitor, i.e., compound **18** or (3~{S})-3-[2-[(dimethylamino)methyl]-1~{H}-indol-3-yl]-5-oxidanyl-2,3-dihydroisoindol-1-one (Molecular formula: C19H19N3O2). Hit compounds demonstrated hydrogen bonds with important residues such as Lys5, Glu37, Ser39, Arg41, Asp54, Gln70, and Thr74. Additionally, both Hit1 and Hit2 demonstrated salt bridge interactions with Glu37. These results thus suggest that the identified compounds can act as RAS isoform inhibitors. The CDOCKER energies and the polar interactions are indicated in the Table S3. However, this would need further validation from molecular dynamics analysis.

**Figure 9:**
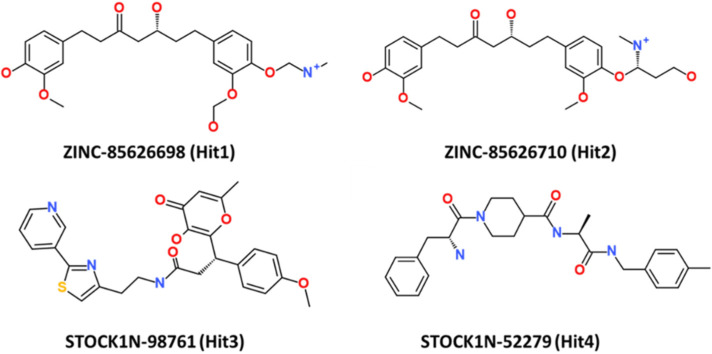
The 2D structure of the four hit compounds.

This research thus provides accounts of computational testing of natural product-based potential inhibitors of RAS isoforms, with a specific emphasis on KRAS G12D. The chemical structures of the four hit compounds, along with their IDs, are provided in Figure 9. 

## 4. Material and Methods

**Figure 10:**
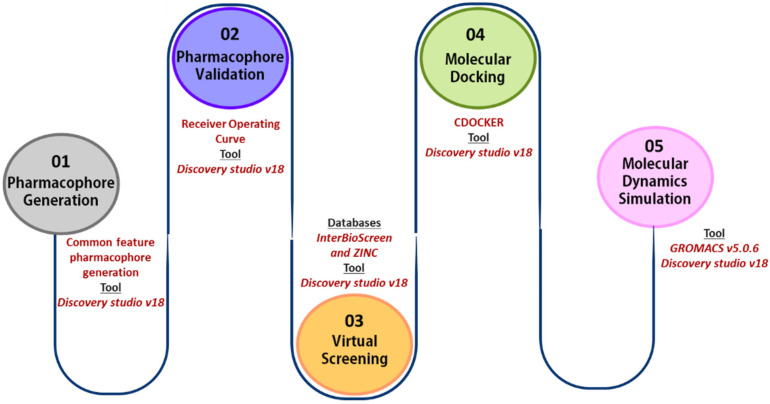
In silico workflow for identification of KRAS G12D inhibitors.

The basic outline of the workflow followed is shown in Figure 10. 

### 4.1. Common Feature Pharmacophore Generation

**Figure 11:**
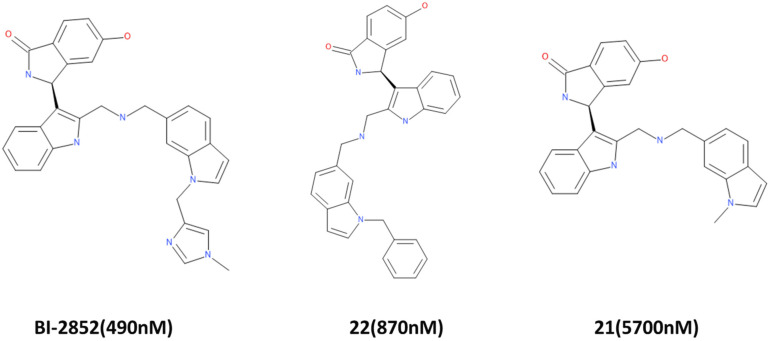
Active compounds for common feature pharmacophore generation. BI-2852 is the most potent and is used as a reference in this study. IC_50_ values are indicated in parentheses.

A training set consisting of in vitro tested KRAS inhibitors from [3] and BindingDB (https://www.bindingdb.org/, accessed on 9 September 2020) [41] was utilized for the generation of common feature pharmacophore. By using the HipHop algorithm [42], the “Common Feature Pharmacophore Generation” tool in Discovery Studio v18 (DS) (Accelrys, San Diego, CA, USA) [43] identifies the key features necessary for the activity of potent inhibitors. The chemical structure of the active ligands considered are shown in Figure 11.

Firstly, important features in these inhibitors were extracted using the “Interaction Generation” tool in DS [43] after which the “Common Feature Pharmacophore Generation” tool in DS was used to generate the model. From the options for the conformation generation “best” was selected, fitting method was set to “flexible” and interfeature distance was fixed at 2.97 Å. All remaining parameters were kept at default. 

### 4.2. Common Feature Pharmacophore Validation

To predict the efficiency of the model to distinguish between active and inactive sets of inhibitors, a validation step was performed. The inactive KRAS inhibitors were obtained from BindingDB [41] with the search term “KRAS” and with IC50 value greater than 6000 nM. Eight inactive inhibitors were identified and their details are provided in Table S1. In total, 11 inhibitors, of which 3 were active and 8 inactive, were employed and a Receiver Operating Characteristic (ROC) curve was generated [44]. ROC classifies the true positivity rate (i.e., sensitivity) from the true negativity rate (i.e., specificity) [45]. The sensitivity and specificity are defined as:

$$Sensitivity=\frac{True\;Positives}{True\;Positives+False\;Negatives}$$

$$Specificity=\frac{True\;Negatives}{False\;Positives+True\;Negatives}$$

The curves are generated simultaneously, along with common feature pharmacophore model generation, by setting validation to “True” in the protocol for the “Common Feature Pharmacophore Generation” tool in DS. 

### 4.3. Virtual Screening

InterBioScreen (https://www.ibscreen.com/) and ZINC (https://zinc.docking.org/) [46] (accessed on 5 October 2020) harboring 69,234 and 144,766 natural compounds, respectively, were screened against the selected pharmacophore. The compounds were filtered for their drug-likeness using “Filter by Lipinski and Veber Rules” with default parameters and then screened for Absorption, Distribution, Metabolism, Excretion, and Toxicity (ADMET) studies by employing the “ADMET Descriptors” tool in DS. Lipinski’s criteria for a compound to be an effective drug candidate states that its molecular weight should be <500 Da, hydrogen bond donors (HBD) should be <5, hydrogen bond acceptors (HBA) should be <10, and an octanol/water partition coefficient (LogP) value should be <5 [47,48]. ADMET parameters such as absorption level = 0 or 1; solubility level = 3 or 4; and blood brain barrier (BBB) = 2 or 3 were chosen [34]. Following the drug-likeness and ADMET steps, the resulting compounds were mapped onto the pharmacophore model by applying the “Ligand Pharmacophore Mapping” tool in DS. Similar to generating the pharmacophore step, the conformer generation and fitting method were set to “best” and “flexible”, respectively.

### 4.4. Molecular Docking

To identify the binding potential of mapped compounds, we performed molecular docking using “CDOCKER” in DS. CDOCKER utilizes CHARMm-based molecular dynamics [34] for performing the molecular docking. In CDOCKER-based molecular docking computations, the receptor is fixed and the ligands are allowed to be flexible [49]. Here, the greater the -CDOCKER interaction energy, the stronger the affinity of the ligand to target [50,51]. Prior to molecular docking, the target structure bearing PDB id: 6GJ8 [3] was cleaned of water and heteroatoms and minimized using the steepest descent algorithm in DS. The ligands were also minimized using the full minimization tool in DS with default parameters. Coordinates amenable to ligand binding of 6GJ8 were obtained by inbound BI-2852 (used as reference in this study). All the parameters of the CDOCKER protocol in DS were set to default. Clusters of the docked poses were generated manually. Based on the CDOCKER interaction energy, binding mode, and molecular interactions, the best pose was selected for each compound from the largest cluster. 

### 4.5. Molecular Dynamics Simulation

To mimic the real-time binding profile of the hit compounds with KRAS G12D, MD simulations were performed using the GROningen MAchine for Chemical Simulations (GROMACS) v5.0.6 package [52]. A total of five systems (Protein-Reference, Protein-Hit1, Protein-Hit2, Protein-Hit3, and Protein-Hit4) were generated for a 100 ns production run. The simulations were run in triplicate. Based on previous studies [53,54,55], a CHARMm27 [56] force field was chosen. SwissParam [57] was used for generating ligand topology. A dodecahedron water box was created around the systems and the TIP3P water model was utilized for solvation. To neutralize the negative charge of the system, Na^+^ ions were added. To remove the steric clashes in the system, steepest decent was chosen for energy minimization. After the system was energy minimized, the protein–ligand complex was restrained followed by equilibration of the system with a constant number *N* of particles, volume *V*, and temperature T (NVT), and constant number N of particles, pressure *P*, and temperature *T* (NPT). Both NVT and NPT ensembles were run at 100 ps, using a V-rescale thermostat for temperature coupling and Berendsen barostat for pressure coupling. Following the volume and pressure equilibrations, the production run was carried out for 100 ns. The “gmx cluster” was used to cluster the poses obtained after the production run. The RMSD cutoff used for “gmx cluster” was 0.1 nm. The representative pose possessing lowest RMSD was selected from the largest cluster. Analysis was performed using gromacs tools and DS.

#### 4.5.1. Root Mean Square Deviation (RMSD) and Root Mean Square Fluctuation (RMSF) Analysis

To determine the effect of ligand binding on the dynamics of protein structure, RMSD and RMSF calculations were performed. The “gmx rmsd” and “gmx rmsf” commands were used to evaluate RMSD and RMSF, respectively.

RMSD is calculated by the following equation [25]:

$$RMSD_{x}=\sqrt{\frac{1}{N}}\sum_{i=1}^{N}\left(r_{i}^{\prime }\left(t_{x}\right)\right)-{\left(r_{i}\left(t_{ref}\right)\right)}^{2}$$

where *N* is the number of atoms, *t_ref_* represents the reference time, *r*′ is the location of the selected atoms within the frame *x* after superimposing on the reference frame, and *t_x_* represents the recoding intervals of *x*.

RMSF is calculated by the following equation [25]:

$$RMSF_{i}=\sqrt{\frac{1}{T}}\sum_{t=1}^{T}\langle \left(r_{i}^{\prime }\left(t\right)\right)-{\left(r_{i}\left(t_{ref}\right)\right)}^{2}\rangle$$

where *T* is the trajectory time, *t_ref_* represents the reference time, *r*′ is the location of the selected atoms within the residue *i* after superimposing on the reference frame, and (< >) is for the average of the square distance taken over residue *i*.

#### 4.5.2. Binding Dynamics and Molecular Interactions

To determine the binding dynamics of hits with KRAS G12D, we took snapshots from the MD run at 0, 25, 50, 75, and 100 ns and superimposed the structures. The “gmx hbond” was used to compute the hydrogen bonds between the proteins and ligands throughout the simulation. In order to access the stability of important interactions, we employed “gmx_distance”. The complete list of molecular interactions between the proteins and ligands were determined by using “Show 2D Diagram” in DS. 

#### 4.5.3. Binding Free Energy

To predict the ligand-binding affinities to the target protein, binding free energy calculations were performed. The Molecular Mechanics/Poisson Boltzmann Surface Area (MM/PBSA) method [58] was used to compute (a) potential energy in vacuum, (b) polar solvation energy, and (c) non-polar solvation energy throughout the 100 ns simulation. The binding free energy of a protein–ligand complex (Δ*G_bind_*) in solution is specified as:

$$\Delta G_{bind}=G_{complex}-\left[G_{protein}+G_{ligand}\right]\;$$

Here, *G_complex_* implies the sum of the free energy of the protein–ligand complex and *G_protein_* and *G_ligand_* imply the free energies of the protein and ligand in their unbound states. 

The solvation term (*G_solv_*) is the combination of the polar (*G_polar_*) and non-polar contribution (*G_nonpolar_*):

$$\Delta G_{solv}=\Delta G_{polar}+\Delta G_{nonpolar}$$

The non-polar contribution (*G_nonpolar_*) is proportional to the solvent accessible surface area (SASA):

$$\Delta G_{nonpolar}=\gamma \left(SASA\right)+\beta \;$$

where *γ* = 0.0227 kJ mol^−1^ Å^−2^ and *β* = 3.849 kJ mol^−1^.

#### 4.5.4. Principal Component Analysis (PCA)

PCA is a useful tool to extract the most useful and meaningful elements (or the principal components) from MD trajectories and would assist in assessing the protein conformational changes upon ligand binding [29]. This was accomplished by calculating eigenvalue and eigenvectors for the covariance matrix, where the eigenvalue represents the magnitude and the eigenvector represents the direction of the motion of the biomolecules during the simulation. The “gmx covar” was used to build and diagonalize the matrix, while the “gmx anaeig” was used to extract the dominant eigenvectors and to calculate the overlap between them and the coordinates of the trajectories.

## 5. Conclusions

Computer-aided drug discovery techniques have greatly assisted in the rapid and cost-effective identification of new drug candidates. In the present research, insilico techniques were employed to identify prospective KRAS G12D inhibitors. A common feature pharmacophore model was generated to extract the important features for KRAS inhibition. ZINC and IBS databases were mapped on the model and mapped compounds were subjected to molecular docking and dynamic simulations. Four potential inhibitors displaying favorable stability with KRAS G12D were obtained. Although all four seem to be promising, substantially better binding free energies to KRAS G12D were obtained with two compounds, ZINC-85626698 and ZINC-85626710. Further experimental validations are warranted to corroborate these computational findings.

## Figures and Tables

**Table 1:** Characteristics of the generated common feature pharmacophore models.

Model No.	Features	Score	Direct Hit	Partial Hit	Max Fit
1	RRHDDA	36.17	11	00	6
2	RRHDDA	36.17	11	00	6
3	RRHDDA	36.17	11	00	6
4	RRHDDA	36.17	11	00	6
5	RRHDDA	36.17	11	00	6
6	RRHDDA	36.17	11	00	6
7	RRHDDA	36.17	11	00	6
8	RRHDDA	36.17	11	00	6
9	RRHDDA	35.96	11	00	6
10	RRHDDA	35.96	11	00	6

**Table 2:** Validation parameters for the common feature pharmacophore models.

Model No.	Total Actives	Total Inactives	True Positives	True Negatives	False Positives	False Negatives	Sensitivity	Specificity
1	3	8	3	5	3	0	1	0.62
2	3	8	3	5	3	0	1	0.62
3	3	8	3	7	1	0	1	0.87
4	3	8	3	5	3	0	1	0.62
5	3	8	3	5	3	0	1	0.62
6	3	8	3	6	2	0	1	0.75
7	3	8	3	4	4	0	1	0.50
8	3	8	3	6	2	0	1	0.75
9	3	8	3	4	4	0	1	0.50
10	3	8	3	7	1	0	1	0.87

**Table 3:** Molecular docking energies of reference and hit compounds.

Compound	-CDOCKER Energy (kcal/mol)	-CDOCKER Interaction Energy (kcal/mol)
**Hit1**	45.6694	53.1082
**Hit2**	35.3224	51.3697
**Hit3**	22.8951	49.2084
**Hit4**	43.3661	49.1899
**Reference (BI-2852)**	25.0164	46.9

**Table 4:** Hydrogen bond occupancy of the hits.

Compound	Hydrogen Bond	Occupancy (%)
**Hit1**	Glu3	88.4
	Gln25	5.8
	Asp54	119
**Hit2**	Asp38	94.9
	Ser39	96.6
**Hit3**	Gln70	14.6
**Hit4**	Gln70	8.6

**Table 5:** Pre and Post MD hydrogen bond interactions between the Hit compounds and KRAS G12D. Distances in nm are given in parentheses.

Compound	Hydrogen Bonds (Molecular Docking)	Hydrogen Bonds (MD Simulations)
**Hit1**	Leu6 (0.30) Glu37 (0.19) Ser39 (0.26) Asp54 * (0.27)	Glu3 * (0.17) Gln25 (0.30) Asp54 * (0.18)
**Hit2**	Glu37 (0.18) Asp54 (0.24) Asp54 (0.26)	Asp38 * (0.15) Ser39 (0.19)
**Hit3**	Lys5 (0.28) Ser39 (0.30) Arg41 (0.33)	Gln70 (0.19)
**Hit4**	Glu37 (0.22) Glu37 (0.24)	Gln70 (0.21)

* Denotes a salt bridge formation.

## Data Availability

Data are contained within the article.

## Supplementary Materials

The following are available online at https://www.mdpi.com/article/10.3390/ijms23031309/s1.
