# Supplementary material for: Identification of New KRAS G12D Inhibitors through Computer-Aided Drug Discovery Methods
Source: Int J Mol Sci. 2022 Jan 24;23(3):1309. doi: 10.3390/ijms23031309 (PMC8836163; doi:10.3390/ijms23031309)
Supplement: Supplementary file 1 [file ijms-23-01309-s001.zip › ijms-1535189-supplementary.pdf]

Identification of New KRAS G12D Inhibitors through Computer- Aided Drug Discovery Methods

Apoorva M Kulkarni [1], Vikas Kumar [1], Shraddha Parate [2], Gihwan Lee [2], Sanghwa Yoon [1] and Keun Woo Lee [1,*]

Table S1. List of inactive compounds from Binding DB for common feature pharmacophore validation.

S. No.	Binding DB Ligand Name	IC50 (nM)
1	CHEMBL3218640	10,000
2	CHEMBL3218639	10,000
3	CHEMBL3218636	10,000
4	CHEMBL4060952	31,000
5	CHEMBL4075783	39,300
6	CHEMBL4074790	65,000
7	CHEMBL4084139	>100000
8	CHEMBL2396992	155,000

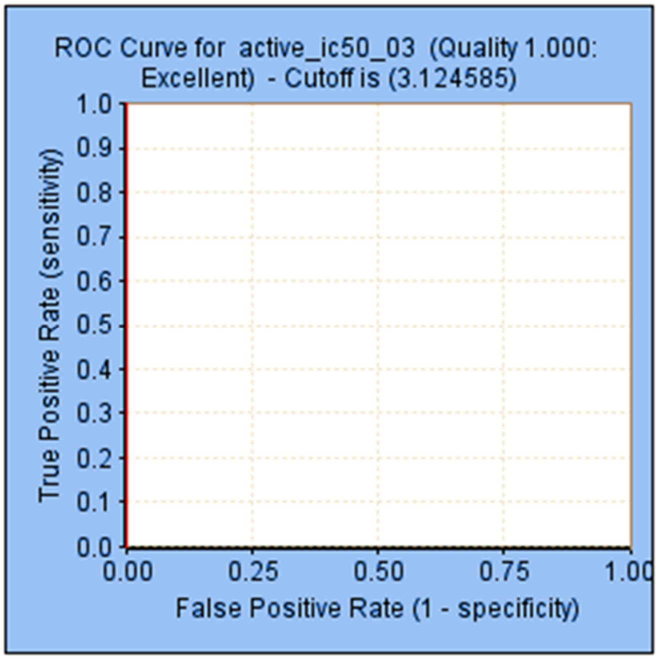

Figure S1. ROC curve of the selected common feature pharmacophore model.

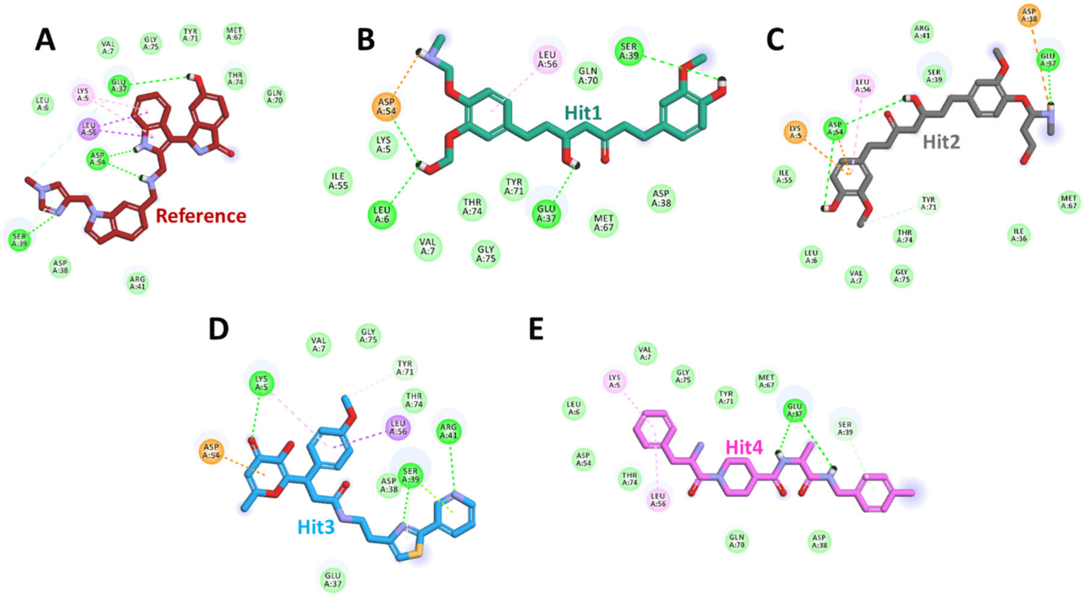

Figure S2. Post molecular docking 2D interactions of reference (**A**) and hit compounds (**B–E**) with KRAS G12D. The reference and the hit compounds are depicted in stick model. Green dashed lines represent hydrogen bond. Residue Asp54 in B participates in salt bridge interactions. All the other dashed lines represent various types of π bonds. Light green colored spheres indicate the residues participating in van der Waals interactions.

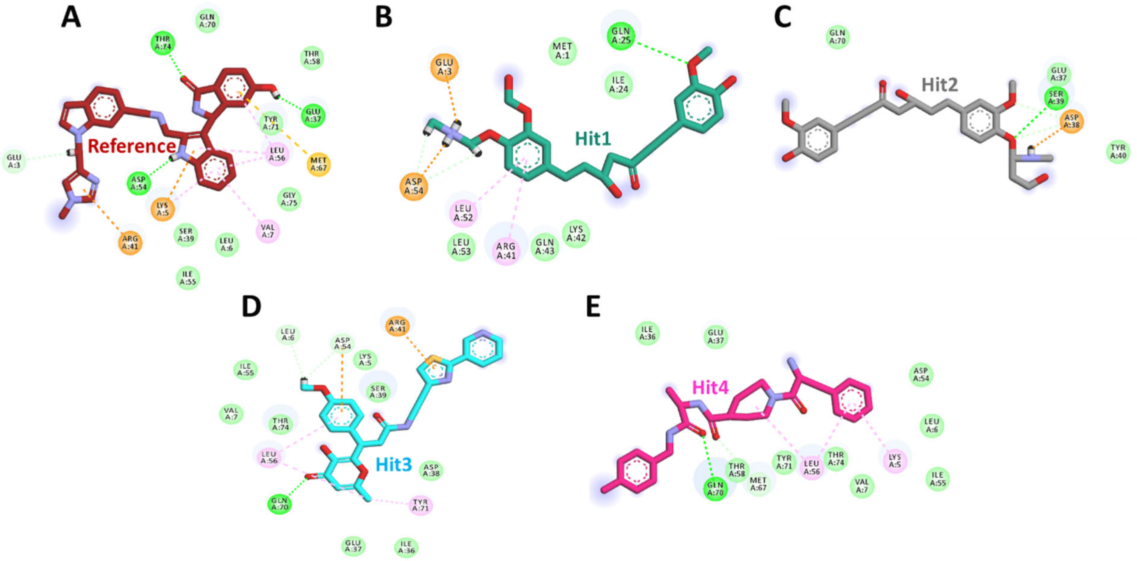

Figure S3. Post MD 2D molecular interactions of reference (**A**) and hit compounds (**B–E**) with KRAS G12D. The reference and the hit compounds are depicted in stick model. Green dashed lines represent hydrogen bond. Residues Glu3, Asp38 and Asp54 in B and C participate in salt bridge interactions. All the other dashed lines represent various types of π bonds. Light green colored spheres indicate the residues participating in van der Waals interactions.

Table S2. ADMET properties of hit compounds.

Compound	Absorption level	BBB level	Solubility level	AlogP	Hydrogen Bond Donors	Hydrogen Bond Acceptors	Mol. Wt (Da)
**Hit1**	0	3	4	1.523	5	8	434.503
**Hit2**	0	3	4	2.013	5	8	462.556
**Hit3**	0	3	3	2.184	2	8	491.559
**Hit4**	0	3	3	2.334	4	7	487.034

Table S3: Molecular docking scores and polar interactions of hit compounds with HRAS and NRAS.

	**HRAS G12D(PDB ID: 6ZJ0)**			**NRAS Q61R (PDB ID: 6ZIZ)**		
**Compound Name**	**- CDOCKER Energy**	**-CDOCKER Interaction Energy**	**Polar Interactions**	**-CDOCKER Energy**	**-CDOCKER Interaction Energy**	**Polar Interactions**
**Hit2**	34.6517	53.3563	Lys5, Leu6, Glu37*	32.4251	49.2873	Glu37*, Arg41
**Hit1**	38.1899	53.2976	Lys5, Glu37*, Ser39(2), Tyr71	36.7247	43.1818	Glu37*, Gln70
**Hit3**	20.0816	42.8491	Lys5, Ser39, Arg41	21.6699	46.2261	Lys5, Arg41, Asp54
**Hit4**	43.2171	43.1558	Lys5, Arg41, Gln70, Thr74	35.6052	35.4795	Glu37, Ser39, Arg41
**BI-2852 (Reference)**	23.5164	42.1177	Lys5, Ser39, Tyr71, Thr74	14.417	30.5704	Glu37, Asp54
**6ZIZ**	16.4809	37.3344	Lys5, Ser39, Asp54, Thr74	8.65547	30.5608	Glu37, Asp54, Thr74

*Denotes salt bridge interaction.
